# Muscle regeneration is disrupted by cancer cachexia without loss of muscle stem cell potential

**DOI:** 10.1371/journal.pone.0205467

**Published:** 2018-10-09

**Authors:** Shoya Inaba, Atsushi Hinohara, Masashi Tachibana, Kazutake Tsujikawa, So-ichiro Fukada

**Affiliations:** 1 Laboratory of Molecular and Cellular Physiology, Graduate School of Pharmaceutical Sciences, Osaka University, Suita, Osaka, Japan; 2 Project for Muscle Stem Cell Biology, Graduate School of Pharmaceutical Sciences, Osaka University, Suita, Osaka, Japan; 3 Immunology & Allergy Research Laboratories, Immunology & Allergy R&D Unit, R&D Division, Kyowa Hakko Kirin Co., Ltd., Nagaizumi-cho, Sunto-gun, Shizuoka, Japan; 4 Business Development Division, Kyowa Kirin Pharmaceutical Research, Inc., La Jolla, CA, United States of America; 5 Project for Vaccine and Immune Regulation, Graduate School of Pharmaceutical Sciences, Osaka University, Suita, Osaka, Japan; 6 Global Center for Medical Engineering and Informatics, Osaka University, Suita, Osaka, Japan; University of Minnesota Medical Center, UNITED STATES

## Abstract

Cancer cachexia is a severe, debilitating condition characterized by progressive body wasting associated with remarkable loss of skeletal muscle weight. It has been reported that cancer cachexia disturbs the regenerative ability of skeletal muscle, but the cellular mechanisms are still unknown. Here, we investigated the skeletal muscle regenerative process in mouse colon-26 (C26) tumor cell-bearing mice as a C26 cancer cachexia model. Although the proliferation and differentiation abilities of muscle stem cells derived from the C26 tumor cell-bearing mice were sustained *in vitro*, the proliferation and differentiation were severely impaired in the cachexic mice. The numbers of both macrophages and mesenchymal progenitors, which are critical players in muscle regeneration, were reduced in the cancer cachexic mice, indicating that the skeletal muscle regeneration process was disrupted by cancer cachexia. Furthermore, the number of infiltrated neutrophils was also reduced in cancer cachexia mice 24 hours after muscle injury, and the expression of critical chemokines for muscle regeneration was reduced in cancer cachexia model mice compared to control mice. Collectively, although the ability to regeneration of MuSCs was retained, cancer cachexia disturbed skeletal muscle regenerative ability by inhibiting the orchestrated muscle regeneration processes.

## Introduction

Cancer cachexia is considered to be a complex syndrome that affects a patient’s life expectancy [1, 2]. The common and important characteristic of cancer cachexia is the loss of skeletal muscle that leads to pronounced body weight loss. It is considered that the wasting muscle condition affects the patient’s survival rate; therefore, the mechanisms underlying cachexia should be better understood in order to treat those patients.

Cancer cachexia weakens the myofiber cell membrane, called the sarcolemma, accompanied by reduced levels of dystrophin, which is the cause of Duchenne muscular dystrophy [3]. In addition, a work conducted by He et al. indicated that cancer cachexia followed by a type of muscle damage resulted in activation of both muscle satellite and non-myogenic cells [4]. Iwata et al. also showed that cancer cachexia causes skeletal muscle damage, although the muscle damage is not severe compared to that of muscular dystrophy in a mouse model [5]. Intriguingly, non-myogenic cells were forced to express Pax7 (a muscle satellite cell marker) through NF-κB signaling in cachexic muscle, which was considered to be one mechanism underlying the impaired muscle regeneration in cachexia model mice [4]. However, detailed analyses of the muscle regeneration processes have remained to be elucidated.

Muscle regeneration absolutely depends on muscle stem cells (MuSCs), also called muscle satellite cells [6–10]. In addition, hematopoietic cells and interstitial cells are also involved in the process of muscle regeneration [11]. When muscle is damaged, neutrophils infiltrate, and then macrophages invade the injured muscle tissue [12, 13]. Neutropenic mice show a severe regeneration defect following reduced macrophage infiltration [14]. Macrophage-depleted mice also showed impaired muscle regenerative processes [12, 13]. The mesenchymal progenitor (also known as fibro/adipogenic progenitors (FAP)) is considered to be a critical interstitial player contributing to the muscle regeneration process [15, 16]. Thus, efficient muscle regeneration requires a well-orchestrated integration of many types of cell.

Here, we showed that the ability to proliferate and differentiate of MuSCs isolated from C26 tumor cell-bearing mice was retained *in vitro*, but the proliferation and differentiation of MuSCs were severely impaired *in vivo* in the cachexic mice. The increase in the numbers of neutrophils, macrophages, and mesenchymal progenitors was disrupted by the cancer cachexia. Our results also show that the expression of critical chemokines for muscle regeneration was reduced in a cancer cachexia model mouse compared to control mice.

## Results

### Reduced muscle weight in cachexia-induced mice

**Fig 1:**
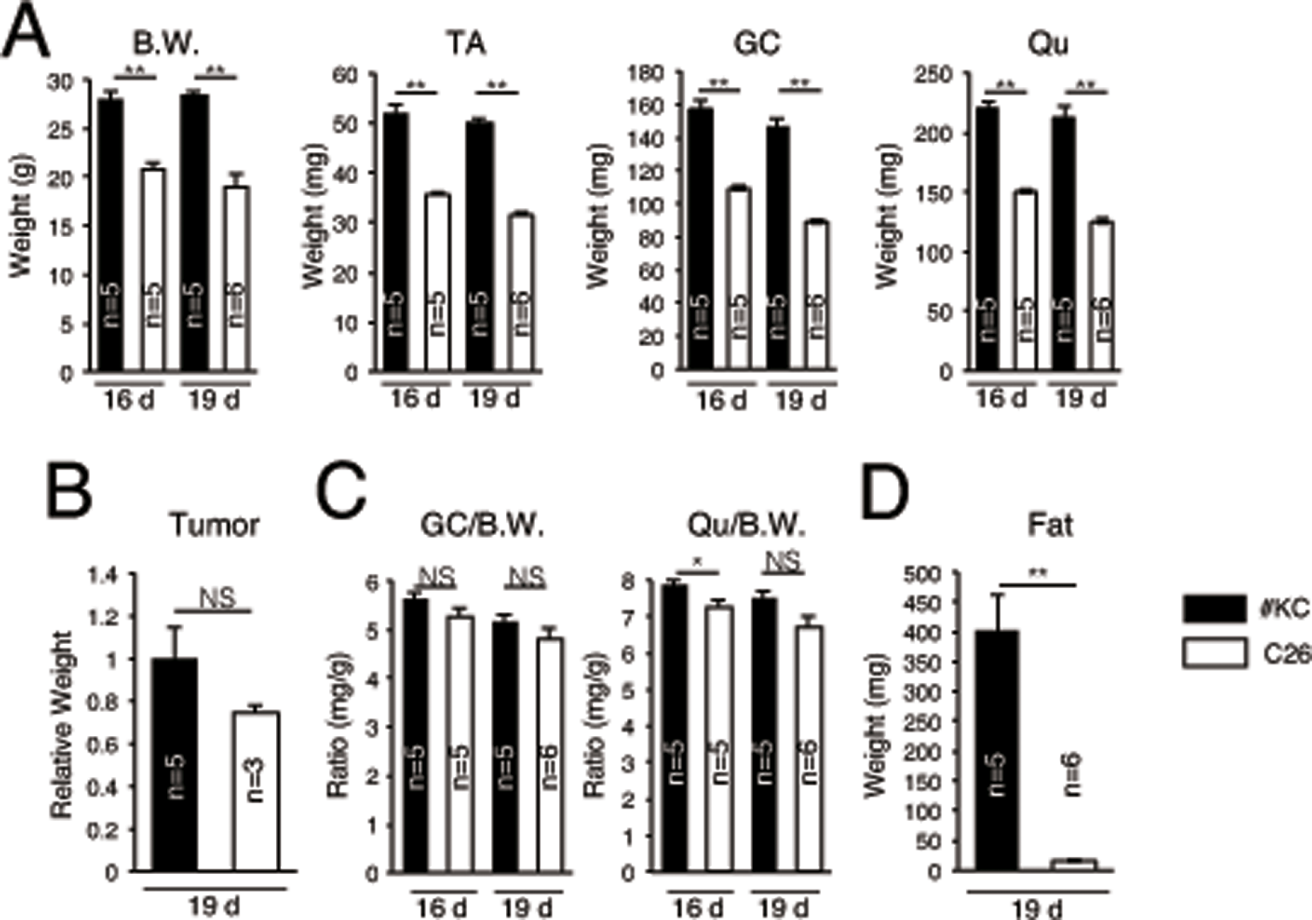
Reduced muscle weight in C26-bearing mice. (A) Body weight (BW), Tibialis anterior (TA), gastrocnemius (GC), and quadriceps (Qu) muscle weights (mg) of #KC (black bar)- or colon26 (C26, white bar)-bearing mice 16 or 19 days after transplantation. (B) Relative tumor weights of #KC (black bar)- and C26 (white bar)- bearing mice 19 days after tumor transplantation. (C) The GC or Qu muscle weights (mg) per body weight (g) of #KC (black bar)- or colon26 (C26, white bar)-bearing mice 16 or 19 days after transplantation. (D) Fat weight (mg) of #KC (black bar)- or C26 (white bar)-bearing mice 19 days after tumor transplantation. *P<0.05, **P<0.01, NS: non-significant.

In this study, we used two colon-26 (mouse colon carcinoma) cell lines. One caused the loss of body weight (hereafter named C26) in mice and the other did not (named #KC) (Fig 1A). The tumor growth of C26 was comparable with that of #KC (Fig 1B). However, 16 or 19 days after C26 or #KC tumor cell implantation, remarkably reduced muscle weights were observed in the limb muscles of C26-implanted mice (Fig 1A). Although there was no significant difference in gastrocnemius (GC) weight per body weight, the result of quadriceps (Qu) weight per body weight also showed the significant difference between C26 and #KC-implanted mice 16 days after the tumor cell implantation (Fig 1C). Like a previous report [17], the weights of fat tissue were also dramatically reduced only in C26-implanted mice (Fig 1D). These results indicated that these models allow us to compare muscle regenerative ability in two tumor-bearing mouse models with or without cachexia phenotypes.

### Severe muscle regeneration defect in cachexia-induced mice

**Fig 2:**
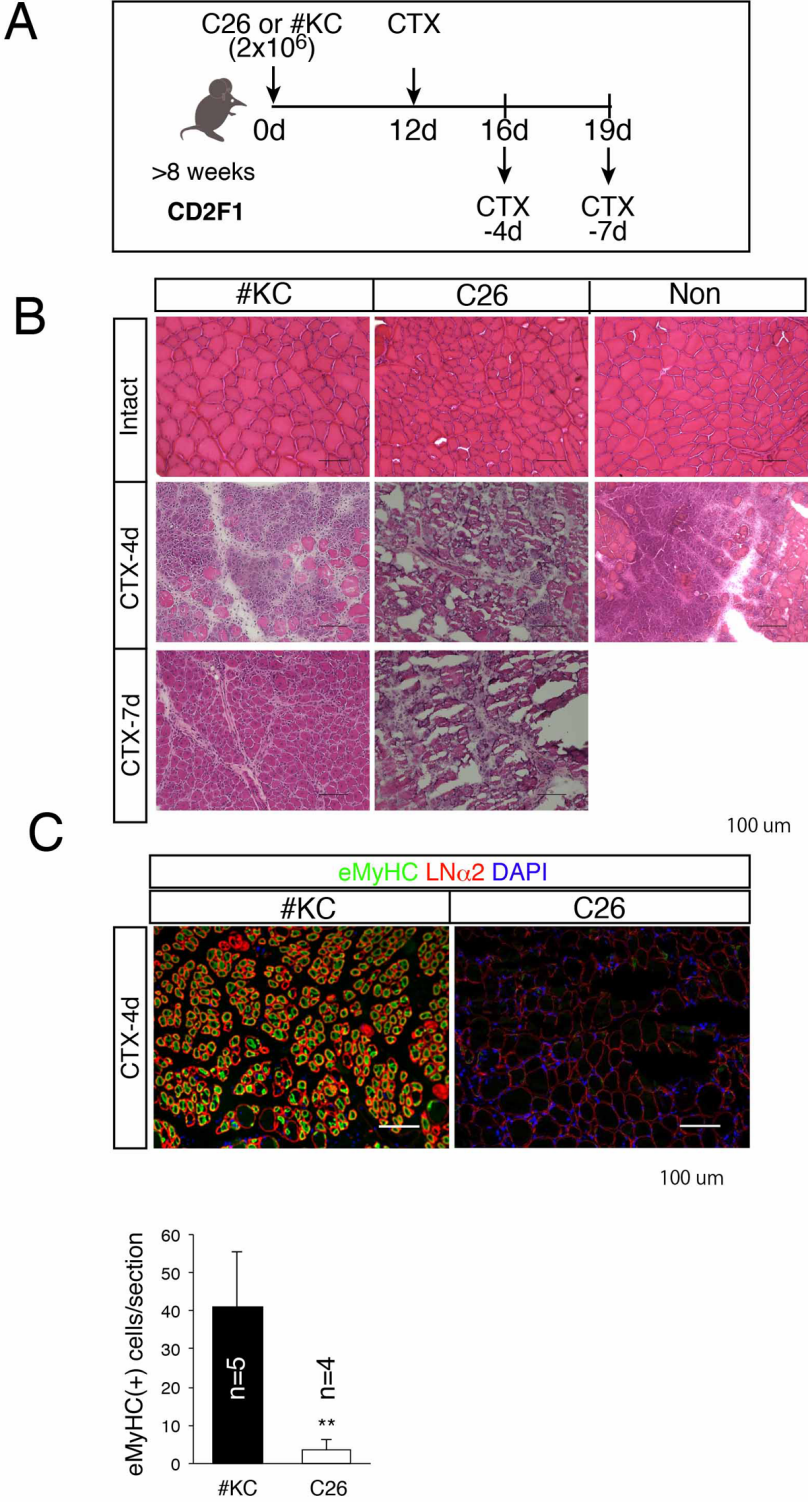
Regeneration defect in C26-bearing mice. (A) Time course for analysis of #KC- or C26-bearing mice. (B) H.E. staining of intact or regenerating TA muscle 4 or 7 days after cardiotoxin injection in #KC-, C26-bearing, or non-transplanted (Non) mice. (C) Immunostaining of eMyHC (green) and laminin α2 (LNα2; red) in regenerating TA muscle 4 days after cardiotoxin injection. Nuclei were counterstained with DAPI. Scale bar: 100 μm. Bar graph shows the percentage of eMyHC-positive areas in #KC- or C26-bearing mice. **P<0.01.

In order to examine the muscle regenerative ability, both C26- and #KC-implanted mice were injected with cardiotoxin (CTX) 12 days after tumor engraftment (Fig 2A). A non-transplanted group was also used in this study. Four to seven days after CTX injection, many mononuclear cells and small myotubes were seen in the #KC group and the non-transplanted group (Figs 2B and S1). In contrast, there were few mononuclear cells in C26-bearing mice. Instead, damaged/necrotic fibers occupied the largest area in the injured muscle of C26-bearing mice (Figs 2B and S1). In uninjured TA muscle, C26-bearing mice had smaller myofibers compared with non-transplanted and #KC groups, indicating that muscle atrophy was induced in the C26-bearing mice (Fig 2B).

Four days after CTX injection, regenerating myofibers marked by embryonic myosin heavy chain (eMyHC) expression were observed in #KC-bearing mice, but not in C26-bearing mice (Fig 2C). Quantitative analyses of the eMyHC area also showed a severe reduction of the regenerative ability in C26-bearing mice (Fig 2C), indicating that muscle regeneration in the cachexia mice was severely disrupted by tumor-induced cancer cachexia, but not tumor non-induced cancer cachexia.

### Overall defect in regeneration process in cachexia-induced mice

**Fig 3:**
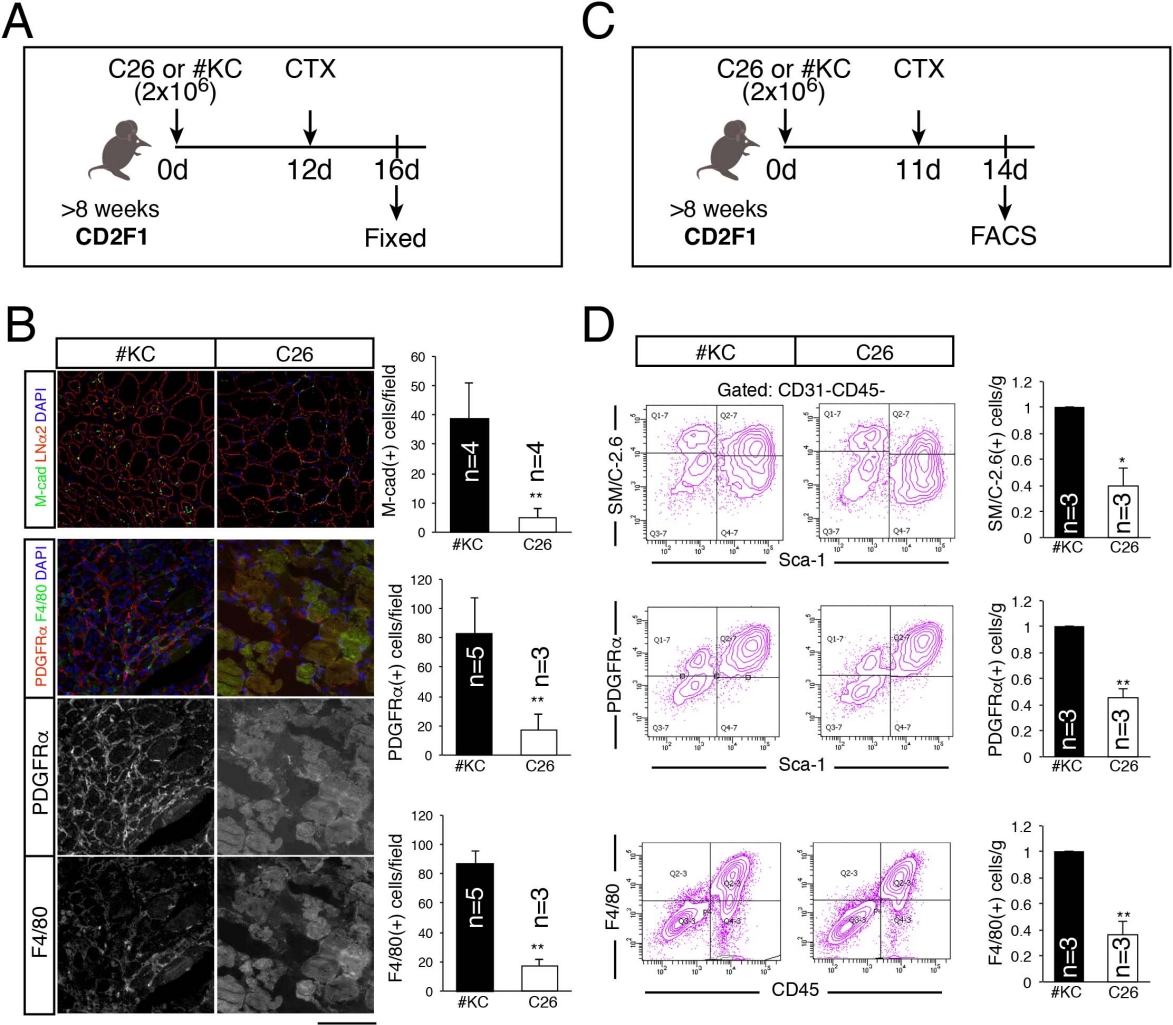
Reduced number of myogenic cells, mesenchymal progenitors, and macrophages in C26-bearing mice during muscle regeneration. (A) Time course of immunohistochemical analyses of #KC- or C26-tumor bearing mice. (B) The sections of TA muscle were stained with anti-M-cadherin (green), laminin α2 (LNα2, red), Pdgfrα (red or white), or F4/80 (green or white) antibodies. Nuclei were stained with DAPI. Scale bar: 100 μm. The graphs indicate the number of each population per field. **P<0.01 (C) Time course of FACS analysis of #KC- or C26-bearing mice. (D) Representative FACS profiles of SM/C-2.6(+)Sca-1(-)CD31(−)CD45(−) cells (myogenic cells), Pdgfrα(+)Sca-1(+)CD31(−)CD45(−) cells (mesenchymal progenitors), and F4/80(+)CD45(+) cells (macrophages) during skeletal muscle regeneration in tumor-bearing mice. TA, GC, Qu muscle were used for these analyses. The graphs indicate the relative number of each population by multiplying the number of mononuclear cell obtained per 1 g muscle and the fraction percentage. *P<0.05, **P<0.01.

In order to reveal which type of cell was most affected by cancer cachexia during muscle regeneration processes, we investigated the numbers of three types of cells, myogenic cells, macrophages, and mesenchymal progenitors, which are essential players in successful muscle regeneration [11]. Injured muscles 4 days after CTX injection were used for the following analyses (Fig 3A). In immunohistological studies, all three types of cells were abundantly present in #KC-bearing mice, but in C26-bearing mice, the cells were rarely detected (Fig 3B). We also did quantitative analyses by flow cytometry (Fig 3C). The FACS profiles of each fraction in C26-bearing mice were similar to those in #KC-bearing mice, but the numbers of cells collected in C26-bearing mice were fewer than that in #KC-bearing mice. Therefore, the cell number per muscle weight (g) calculated by FACS showed results similar to the immunohistological results (Fig 3D). Taken together, those results indicate that muscle regeneration processes were globally, rather than a particular type of cells, suppressed in C26-bearing mice.

### Regeneration ability of muscle satellite cells was sustained in cachexia-induced mice

**Fig 4:**
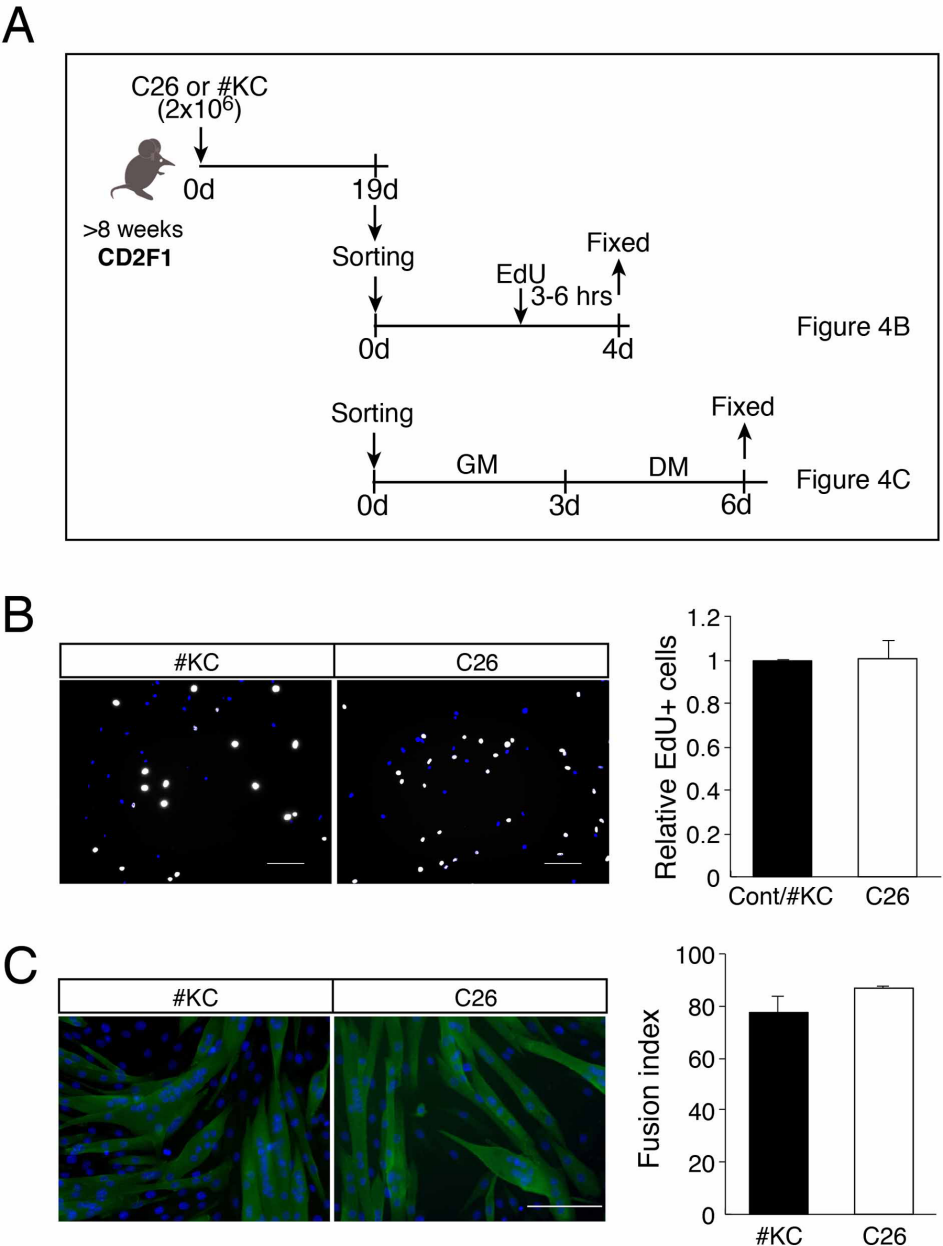
Isolated myogenic cells from C26-bearing mice show normal proliferation and differentiation. (A) Time course of EdU uptake (B) or fusion index (C) of MuSCs derived from TA, GC, Qu muscles of #KC- or C26-bearing mice. (B) Percentage of EdU+ cells (white) in cultured myogenic cells from #KC- or C26-bearing mice. Scale bar: 100 μm. The graph shows the relative number of EdU+ cells per total number of nuclei. (C) Index of the frequency of fusion in freshly isolated myogenic cells from #KC- or C26-bearing mice. Myotubes were stained with anti-α-actinin (green). Scale bar: 100 μm. The graph shows the percentages of multinuclear cell per total number of nuclei.

Skeletal muscle cannot regenerate in a damaged area when MuSCs are absent [6, 7]. MuSCs were isolated from the intact muscle and cultured in an activated and proliferating state *in vitro*. Using these MuSCs, we compared the proliferative and differentiative abilities of MuSCs derived from C26-bearing mice with those of #KC-bearing mice (Fig 4A). As shown in Fig 4B, the EdU uptake of C26-bearing mouse-derived MuSCs was comparable with that of MuSCs derived from #KC-bearing mice, indicating that the proliferation capacity of MuSCs in C26 was sustained even in the cancer cachexia condition.

We next examined the differentiation potential *in vitro*. As shown in Fig 4C, the MuSCs derived from C26-bearing mice differentiated normally and generated multinuclear myotubes like the #KC-bearing mouse-derived MuSCs. Taken together, the proliferation and myogenic differentiation abilities of MuSCs were sustained in the cancer cachexia muscle.

### Neutrophil infiltration was decreased in cachexia-induced mice

**Fig 5:**
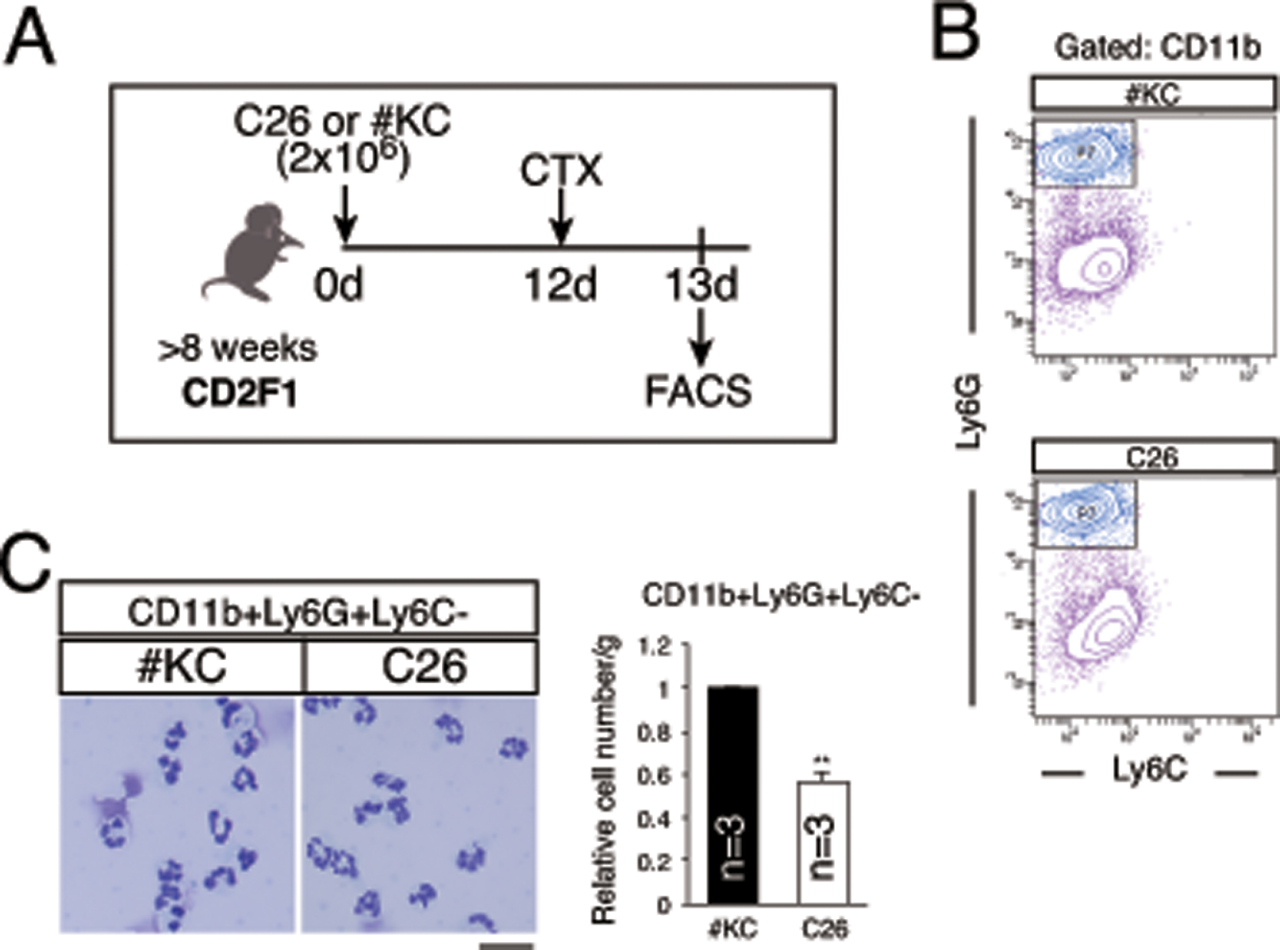
Severe regeneration defect in C26-bearing mice. (A) Time course of FACS analysis of neutrophils derived from #KC- or C26-bearing mice. (B) FACS profiles of mononuclear cells derived from #KC- (upper) or C26-bearing (lower) TA, GC, Qu muscles 1 day after CTX injection. The cells were gated for CD11b+ fractions. (C) Giemsa staining of CD11b+Ly6G+Ly6C- fractions. Scale bar: 20 μm. The graph shows the relative cell numbers of CD11b+Ly6G+Ly6C- fractions per 1 g muscle. **P<0.01.

To further examine the impaired muscle regeneration in the cachexic mice, we focused on neutrophils. Prior to MuSC proliferation and macrophage accumulation, neutrophils infiltrate into damaged muscle [12]. Using anti-CD11b, Ly6G, and Ly6C antibodies, we analyzed mononuclear cells derived from C26- and #KC-bearing mice one day after CTX injection (Fig 5A). Neutrophils were characterized as CD11b+Ly6G+Ly6C- cells (Fig 5B); in fact, the cells in this fraction showed the representative neutrophil nuclear shape and polymorphic nuclear structure (Fig 5C). Like the results of macrophages and mesenchymal progenitors (Fig 3D), the percentage of neutrophils in FACS profiles of C26-bearing mice was similar to that of #KC-bearing mice, but the total cell number collected in C26-bearing mice was fewer than that in #KC-bearing mice. Therefore, the absolute number of infiltrated neutrophils in C26-bearing mice was fewer than that in #KC-bearing mice (Fig 5C).

### Chemokines for infiltration of neutrophils were reduced in cancer cachexia muscle

**Fig 6:**
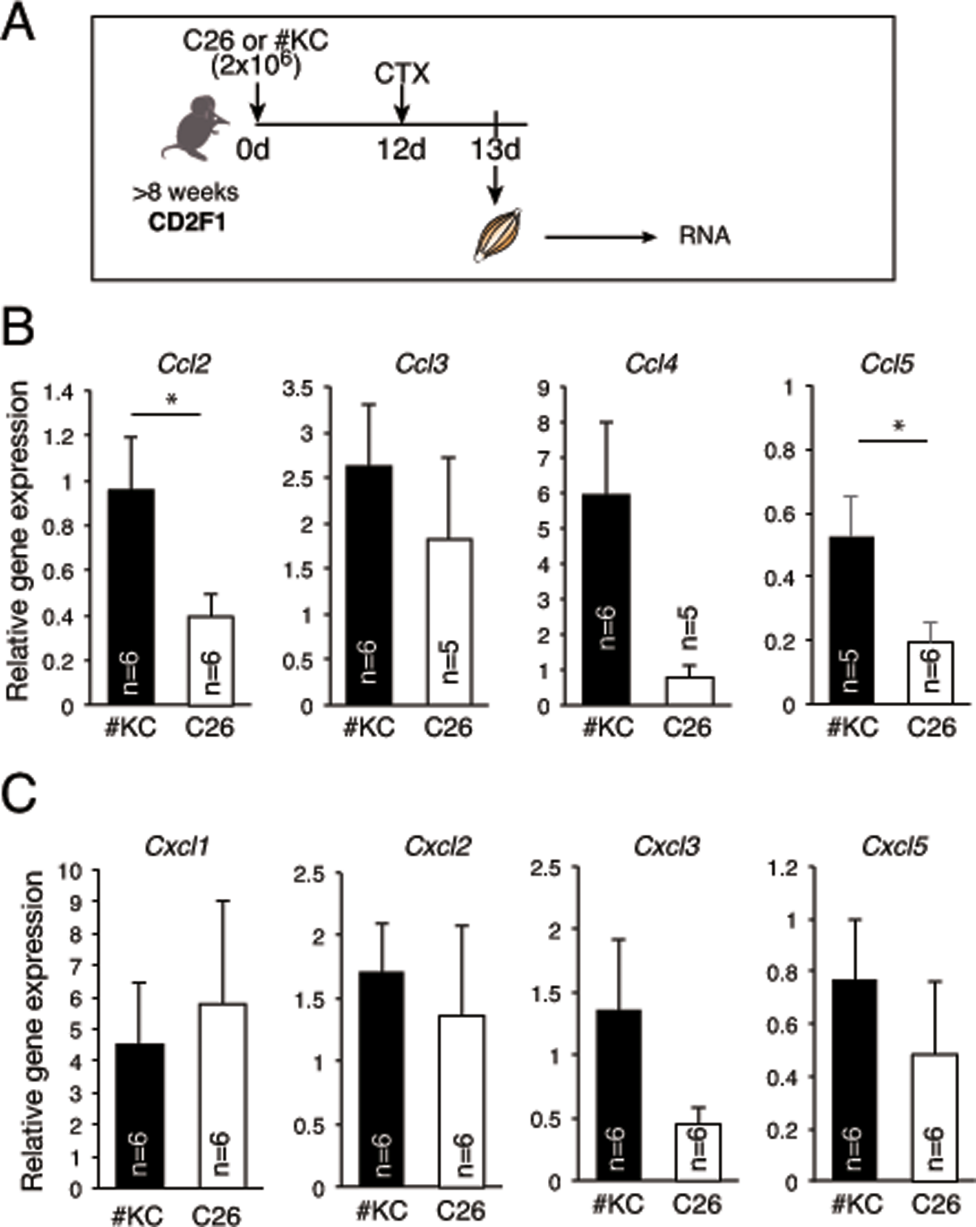
Expression of chemokines inducing neutrophil infiltration. (A) Time course of mRNA expression of chemokines in #KC- or C26-bearing muscle 1 day after CTX injection. (B) Relative expressions of Ccl2-5 mRNA in injured TA, GC, Qu muscle from #KC (black)- or C26 (white)-bearing mice 24 hours after CTX injection. *P<0.05 (C) Relative expressions of Cxcl1-3, 5 mRNA in injured muscle 24 hours after CTX injection from #KC (black)- or C26 (white)-bearing mice.

Finally, we examined the mechanism of why neutrophil and macrophage infiltration was reduced in the cachexic mice. Chemokines are essential for neutrophil and macrophage infiltration. The representative chemokines for macrophage migration into damaged muscle are the CC family. Experiments using Ccl2-knock-out mice especially showed that Ccl2 expression is necessary for the repair of injured skeletal muscle [18]. In addition, Ccl3 and Ccl4 expressions were also dramatically increased one day after muscle injury [19]. Therefore, we examined the expressions of Ccl2–5 in injured muscle one day after CTX injection (Fig 6A), and found that the expressions of these chemokines were significantly reduced or tended to be reduced in C26-bearing mice compared to #KC-bearing mice (Fig 6B).

In general, CXC family chemokines attract polymorphonuclear neutrophils [20]. Therefore, we also examined whether the expressions of Cxcl1-3,5 were affected by cancer cachexia. As shown in Fig 6C, there was no significant difference, but the expression of Cxcl3 in C26-bearing mice showed a tendency to decrease in expression. The detailed mechanism of the reduced infiltration of neutrophils is the remaining question, which might lead to understanding of the effect of cancer cachexia on skeletal muscle.

## Discussion

### Cancer cachexia, damaged muscle, and muscle satellite cells

As previously mentioned, weakness and instability of the sarcolemma have been reported by independent research groups [3, 5, 21]. In both muscles of human gastric cancer patients and mouse of a mouse cancer cachexia model, Pax7 and MyoD mRNA/protein levels were upregulated [4, 22]. Because activated MuSCs are positive for both Pax7 and MyoD [23], these results suggest that the MuSC may be in an activated state. In fact, Penna et al. showed that the MuSC number was increased in uninjured muscle by cancer cachexia using anti-Pax7 or caveolin-1 antibody [24]. He et al. also showed increased numbers of Pax7+ cells in cachexia muscle [4]. Here, we observed the tendency of MuSC number to increase by using a different MuSC-specific marker, M-cadherin staining (S2 Fig). Collectively, those results suggest that MuSCs became activated and proliferated in cancer cachexia muscle. However, to our knowledge, there is no direct evidence showing that cancer cachexia induces substantial damages in myofibers of both human patients and mouse models. Neither did we detect damaged myofibers in our C-26 mice model. A specific microenvironment is critical for maintaining MuSCs [25]. In contrast, the impaired equilibrium leads to the activation or loss of the MuSC pool [10]. The sarcolemma structure is affected by cancer cachexia [3], which might disturb the quiescence of MuSCs, or circulating factor(s) derived from cancer affected the quiescent state of MuSCs in a direct or indirect manner. Understanding in detailed the mechanism of the increased number of MuSCs might lead to elucidate the maintenance mechanism of MuSCs in a steady condition.

### Impaired muscle regeneration in cancer cachexia muscle

There are several reports on the effect of cachexia on muscle regeneration [21, 26]. To investigate the proliferation of MuSCs, He et al. labeled proliferating myogenic cells with BrdU and examined the number of BrdU+ myonuclei derived from proliferated MuSCs [4]. Because the number of BrdU+ myonuclei in C26-bearing muscle was decreased, the data suggested that the proliferation of MuSCs in cachexia muscle in vivo was impaired. However, there was a differentiation defect in the myogenic cells of cachexia muscle [4], meaning that to what extent cancer cachexia affects the proliferation of MuSCs was unclear. In addition, it was unclear whether MuSCs have a proliferation defect in vitro or not. Here, our data clearly showed that the proliferation of MuSCs was severely decreased by cancer cachexia in vivo. In contrast, their proliferative ability was retained in vitro, indicating that the ability of MuSCs was not altered irreversibly, but that MuSCs sustain their original ability to proliferate.

Regarding the differentiation ability of MuSCs in cancer cachexic muscle, He et al. reported that myogenic cells exposed to cachectic factors retained the capacity to differentiate similar to our proliferation results, because the cells isolated from tumor-bearing mice showed a greater fusion index compared with controls [4]. Our data also supports their observation. On the other, Penna et al. pointed out that the increased phosphorylated myogenin level observed in cancer cachexic muscle might be a putative mechanism for the impaired muscle regeneration response in cancer cachexic muscle [24]. As aforementioned, the proliferative ability of MuSCs-derived from C26-bearing mice was sustained, suggesting the phosphorylated myogenin level might be reversible in MuSCs of cachexic muscle.

TNF-α is a candidate to inhibit myogenic differentiation, but Pax7 expression was not regulated by TNF-α [4]. MyoD mRNA was reduced by TNF-α [27], but the MyoD mRNA level was increased in cancer cachexia muscle [22]. In addition, the TNF-α mRNA level was increased during normal muscle regeneration, and the pro-myogenic action of TNF-α was also reported [28]. Collectively, TNF-α seemed not to be a cytokine suppressing Pax7 and myogenic differentiation in cancer cachexia.

A new finding of our present study is that overall regeneration processes, but not a particular type of cell, were affected by cancer cachexia. The first cellular event in muscle regeneration, the infiltration of neutrophils, was disrupted in C26-bearing mice. Among the chemokines tested here, Ccl2 and Ccl5 mRNA expressions were significantly reduced in cachexic mice. The Ccl2 deficiency blocked monocyte/macrophage recruitment into injured muscles [18]. On the other hand, the Ccl2 deficiency did not affect the infiltration of neutrophils 24 hours after muscle injury [18]. These results suggest that the reduced number of infiltrated macrophages might result from the reduced expression of Ccl2, and that the decreased number of infiltrated neutrophils was not explained by the reduced expression of Ccl2. In the muscle regeneration model, the inhibition of neutrophil infiltration results in impaired muscle repair, suggesting that one primary effect of cancer cachexia was impairment of neutrophil infiltration. Of course, we cannot deny the direct effect of cancer cachexia on macrophages and mesenchymal cells. In fact, He et al. indicated the myogenic conversion of mesenchymal progenitors upon Pax7 expression [4].

The decreased number of macrophages and myogenic cells might be explained by the reduced infiltration of neutrophils, because the infiltration of macrophages or the proliferation of myogenic cells are regulated by neutrophils or macrophages, respectively. On the other hand, the detailed mechanism of the reduced number of mesenchymal progenitors is largely unknown. In general, mesenchymal progenitors proliferate profusely and differentiate to fibroblastic cells under pathogenic and impaired muscle regeneration conditions. Therefore, we speculate that, in cachexia, the muscle regeneration processes are not merely orchestrated, but certain factor(s) actively inhibit the processes including the proliferation of mesenchymal progenitors. Although it has been unclear whether the factor that inhibits regeneration also leads to muscle atrophy or not, identification of the factor that inhibits regeneration might elucidate the mechanism underlying the induction of muscle atrophy by cancer cachexia.

In conclusion, cancer cachexia dramatically inhibits muscle regeneration processes. Although the capacity of MuSCs to regenerate was retained in the cachexic condition, they did not proliferate and differentiate efficiently. One mechanism is the reduced number of neutrophils, macrophages, and mesenchymal progenitors, a part of which might be explained by the reduced expression of chemokines. It is important to identify the inhibitor of regeneration to deepen understanding of the mechanism underlying the loss of skeletal muscle in cancer cachexia.

## Materials and methods

### Colon cell lines

C26 tumor cells were obtained from Kyowa Hakko Kirin Co., Ltd. #KC tumor cells (TKG0518) were obtained from the Cell Resource Center for Biomedical Research, Tohoku University. Both cell lines were cultured in RPMI 10% FCS containing penicillin and streptomycin.

### Mouse cachexia model

Six- to eight-week-old CD2F1/Crlj (CD2F1) mice were purchased from Nihon Charles River (Yokohama, Japan). Three animals were housed in one cage designed for six mice and maintained in a controlled environment (temperature of 24±2°C, humidity of 50 ± 10%) with a 12:12 h light;dark cycle. The mice received sterilized standard chow (DC-8, Nihon Clea, Tokyo, Japan) and water ad libitum. Animal health was monitored in accordance with the recommendations of the Guide for the Care and Use of Laboratory Animals (the Japanese Society for Laboratory Animal Resources). In order to alleviate suffering, C26 or #KC tumor cells (2 x 10^6^ cells per mouse) were injected into the right flanks of mice under the use of isoflurane anesthesia. All efforts were made to alleviate suffering during all experiments. A humane endpoint was determined according to the criteria of the Japanese Society for Laboratory Animal Resources. At the end of the planned experiments, the mice were immediately euthanized with overdose pentobarbital. All procedures for experimental animals were approved by the Experimental Animal Care and Use Committee at Osaka University (approval number; 25-9-3).

### Muscle injury model

In order to induce muscle regeneration, 50–100 μL of cardiotoxin (CTX) from *Naja mossambica mossambica* (10 μM in PBS, Catalog number C9759-5MG, Sigma-Aldrich, St. Louis, MO, USA) or CTX from *Naja pallida* (Latoxan, France) was injected into tibialis anterior (TA) muscles. For FACS analyses, tibialis anterior (TA), gastrocnemius (GC), and quadriceps (Qu) muscles were damaged by CTX.

### Measurement of adipose tissues

When mice were sacrificed, their epididymal adipose tissue was harvested and weighed.

### Muscle fixation and histological analysis

Isolated tibialis anterior muscles were frozen in liquid nitrogen-cooled isopentane.

(Wako Pure Chemicals Industries). Transverse cryosections (10 μm) were stained with H&E.

### Preparation and FACS analyses of skeletal muscle-derived mononuclear cells

TA, GC, and Qu muscles were used in this study. Mononuclear cells from uninjured or injured limb muscles were prepared using 0.2% collagenase type II (Worthington Biochemical) as previously described [29]. FITC-conjugated anti-CD31, -CD45, PE-conjugated anti-Sca-1, and biotinylated-SM/C-2.6 [30] antibodies were used for satellite cell staining.

For detection of macrophages or neutrophils, FITC-conjugated anti-CD45 and PE-conjugated anti-F4/80 (Clone; BM8, BioLegend) or PE-conjugated anti-CD11b (Clone; M1/70, BD Pharmingen), APC-conjugated anti-Ly6G (Clone; 1A8, BioLegend), and V450-conjugated anti-Ly6C (Clone; AL-21, BD Pharmingen) antibodies were used, respectively. For detection of mesenchymal progenitors, FITC-conjugated anti-CD31, -CD45, PE-conjugated anti-Sca-1, and biotinylated anti-PDGFRα (R&D Systems, Minneapolis, MN, USA) were used as described previously [16]. Cell sorting was performed using an FACS Aria II flow cytometer (BD Immunocytometry Systems).

### Immunohistological staining

Transverse sections (7 μm) of muscles were reacted with anti-laminin α2 (clone: 4H8-2, Alexis Biochemicals, San Diego, CA, USA), anti-PDGFRα (R&D Systems), anti-F4/80 (Clone: A3-1, Abcam), embryonic myosin heavy chain (eMyHC, clone: F1.652, Developmental Studies Hybridoma Bank, Iowa City, IA, USA), or anti-M-cadherin antibodies [31]. After the first staining at 4°C overnight, sections were incubated with a secondary antibody conjugated with Alexa 488 or 546 (Molecular Probes, Eugene, OR, USA). Coverslips were mounted using Vectashield (Vector Laboratories, Inc., Burlingame, CA, USA). The signals were recorded photographically using a BZ-X700fluorescence microscope (Keyence).

### Immunocytochemistry (EdU and fusion index)

For EdU detection, freshly isolated muscle satellite cells were cultured for 3–4 days in growth medium (GM) (DMEM-HG containing 20% FCS (Trace Biosciences, N.S.W., Australia), 2.5 ng/ml basic fibroblast growth factor (bFGF) (FGF2:PeproTech, London, UK), and penicillin (100 U/ml)-streptomycin (100 μg/ml) (Gibco BRL, Gaithersburg, MD, USA)) on culture dishes coated with Matrigel (BD Biosciences). Thirty-six hours before fixation, EdU was added to the GM medium. The cultured cells were fixed with 4% PFA for 10 minutes and then permeabilized with 0.25% Triton X-100 in PBS for 20 minutes. The Click chemical reaction was performed according to the manufacturer’s instructions using a Click-iT EdU Imaging Kit (Invitrogen, Carlsbad, CA, USA). Coverslips were mounted using Vectashield (Vector Laboratories).

For fusion index analyses, freshly isolated muscle satellite cells were cultured for 3 days in GM on culture dishes coated with Matrigel (BD Biosciences). Then the medium was changed to differentiation medium containing DMEM-HG, 5% horse serum, and penicillin-streptomycin for 3 days. The cultured cells were fixed with 4% PFA as described above. After blocking with 5% skimmed milk, the cells were stained with anti-sarcomeric α-actinin antibody (Sigma-Aldrich, clone EA-53).

### RNA isolation and RT-PCR analysis

Total RNA was extracted from TA muscles using Qiagen Tissue Ruptor Disposable Probes (Nonsterile) (Cat: 990890), Qiagen Tissue Ruptor (Cat: 9001271), and a Qiagen RNeasy Fibrous Tissue Mini Kit (Cat: 74704) according to the manufacturer’s instructions (QIAGEN) and then reverse-transcribed into cDNA using a QuantiTect Reverse Transcription Kit (QIAGEN). Specific forward and reverse primers for optimal amplification in real-time PCR of reverse transcribed cDNAs are listed in S1 Table.

### Statistics

Values were expressed as means±SE. Statistical significance was assessed by a two-tailed Student’s t-test. In comparisons of more than two groups, non-repeated measures analysis of variance (ANOVA) followed by the Bonferroni test (versus control) or SNK test (multiple comparisons) were used. A probability of less than 5% (p<0.05) or 1% (p<0.01) was considered statistically significant.

## Supporting information

S1 Fig Regeneration of non-implanted and C26-bearing mice. H.E. staining of regenerating TA muscle 4 or 6 days after cardiotoxin injection in non-implanted or C26-bearing mice.
(EPS)

S2 Fig Muscle satellite cells number in C26-bearing mice. M-cadherin+ cells number per 100 myofibers in non-injured TA muscle of non-implanted or C26-bearing mice.
(EPS)

S1 Table Primer list. (DOCX)
