# Supplementary material for: Muscle regeneration is disrupted by cancer cachexia without loss of muscle stem cell potential
Source: PLoS One. 2018 Oct 9;13(10):e0205467. doi: 10.1371/journal.pone.0205467 (PMC6177180; doi:10.1371/journal.pone.0205467)
Supplement: S1 Table — (DOCX) [file pone.0205467.s003.docx]

| **Supplemental Table I: Primer list** | | | |
| --- | --- | --- | --- |
| Gene |  | Sequence | Size (bp) |
| Ccl2 | Fwd | agttaacgccccactcacc | 147bp |
|  | Rev | gcacagacctctctcttgagc |  |
| Ccl3 | Fwd | cctgctgcttctcctacagc | 197bp |
|  | Rev | ctgcctccaagactctcagg |  |
| Ccl4 | Fwd | atgaagctctgcgtgtctgc | 214bp |
|  | Rev | gtctgcctcttttggtcagg |  |
| Ccl5 | Fwd | ctcaccatcatcctcactgc | 184bp |
|  | Rev | tccttcgagtgacaaacacg |  |
| Cxcl1 | Fwd | acccaaaccgaagtcatagc | 119bp |
|  | Rev | ctccgttacttggggacacc |  |
| Cxcl2 | Fwd | agtgaactgcgctgtcaatg | 153bp |
|  | Rev | ttcagggtcaaggcaaactt |  |
| Cxcl3 | Fwd | cagtgcctgaacaccctacc | 127bp |
|  | Rev | ggcaaacttcttgaccatcc |  |
| Cxcl5 | Fwd | cgctaatttggaggtgatcc | 193bp |
|  | Rev | aacactggccgttctttcc |  |
